# Comparison of oscillatory wall shear stress in the abdominal aorta of men and women: relationship to abdominal aortic aneurysm (AAA) development

**DOI:** 10.1186/1532-429X-18-S1-O21

**Published:** 2016-01-27

**Authors:** Elizabeth Iffrig, William R Taylor, John Oshinski

**Affiliations:** 1 Cardiology, Emory University, Atlanta, GA USA; 2 Biomedical Engineering, Georgia Institute of Technology, Atlanta, GA USA; 3 Radiology, Emory University, Atlanta, GA USA; 4 School of Medicine, Emory University, Atlanta, GA USA

## Background

Numerous animal and human studies have confirmed a relationship between inflammation, wall shear stress (WSS, the frictional force of fluid felt at a vessel wall) and vascular pathologies. These studies suggest the importance of WSS as a predictive factor in evaluating an individual's risk of developing vascular disease. There have been several observational studies which indicate that men are four times more likely to develop abdominal aortic aneurysm (AAA) than women. Based on the known link between oscillatory WSS and aneurysm dilation, we conducted a study to investigate differences in the WSS patterns in the abdominal aortas of men and women. We utilized a method of measuring WSS which combines an analytical solution to the Navier-Stokes equations and phase contrast magnetic resonance (PCMR) derived flow waveforms. The hypothesis was that women would have a lower values of pro-inflammatory oscillatory WSS than men.

## Methods

We enrolled 13 women and 9 men aged 30-42 with no history of cardiovascular disease. We acquired a non-contrast MR angiogram of the abdominal aorta for each volunteer as well as multiple 2D PCMR scans along the length of the abdominal aorta, perpendicular to the primary flow direction (7 ± 2 locations on average, velocity encoded in the through plane direction). Using a method developed in our lab, we quantified flow waveforms at each slice location from the PCMR data and computed time-dependent WSS based on regional flow constrained to the Wormersley solution to the Navier-Stokes equation. We evaluated forward and reverse flow for the nearest 25% of the vessel, and oscillatory shear stress index (OSI, the net shear in reverse direction normalized to the total net shear) at 80 locations around the circumference of the vessel. The data values in the aorta were ‘unwrapped' and plotted in a matrix of longitudinal (along aorta) versus circumferential (around aorta). Data was grouped by sex and averaged over subjects at each point in the matrix. Using a t-test, we compared the average values over the whole aorta and at each location for reverse flow, forward flow, and OSI.

## Results

**Figure 1:**
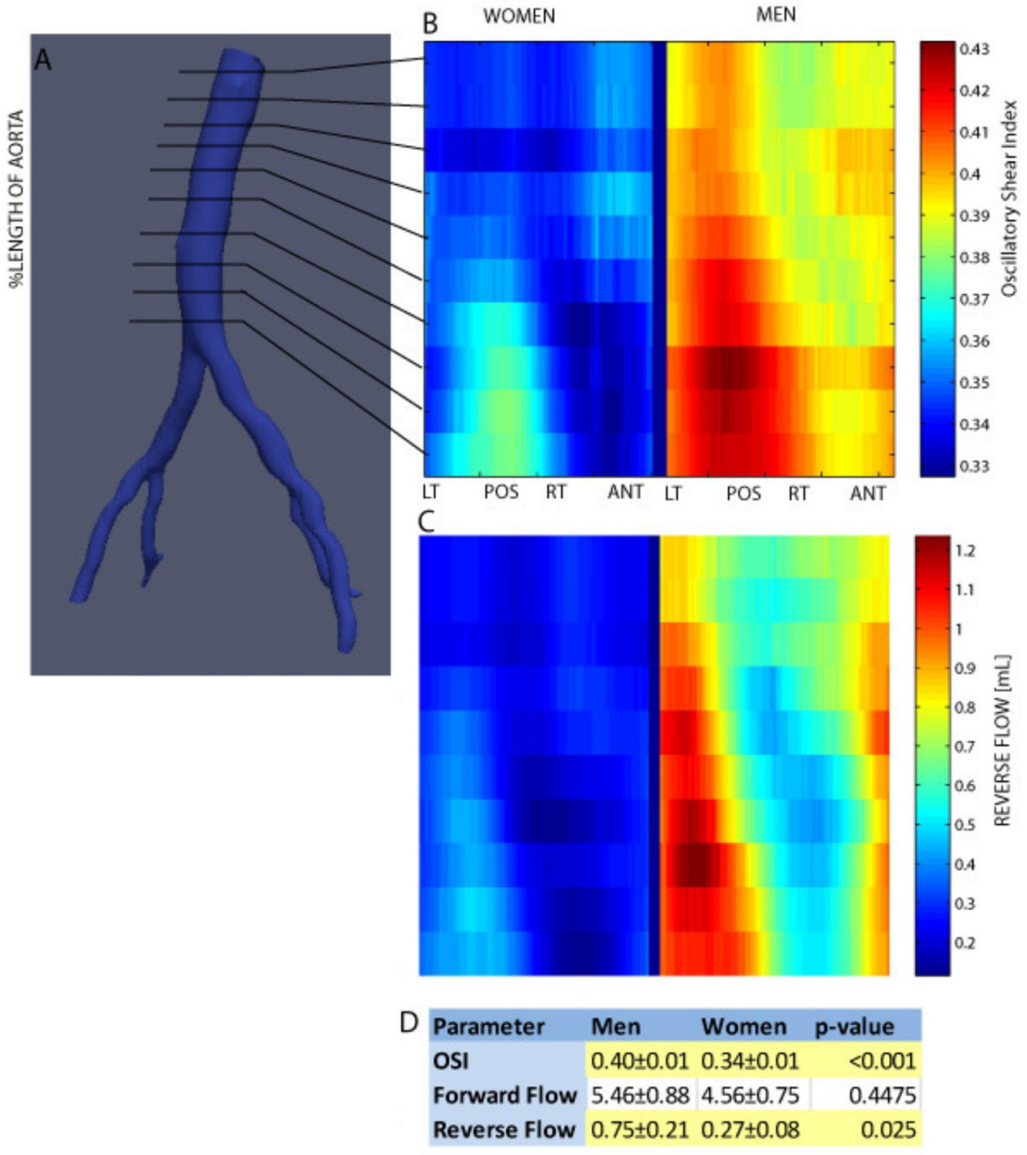
Figure 1

Averaged over the entire abdominal aorta, we found significant differences in both reverse flow and OSI between men and women, but no difference in forward flow. The results of this are summarized in the table in Figure 1D. At each location in the abdominal aorta, men had significantly higher OSI than women (p < 0.05 for every location in the matrix). OSI tended to increase distally along the aorta, most notably on the posterior of the vessel.

## Conclusions

Using a new method to quantify WSS from PCMR, we were able to demonstrate differences in the OSI between men and women, *most notably that men have a higher degree of oscillatory shear when compared with women at all location in the abdominal aorta*. Given the pro-inflammatory character of oscillatory shear, we propose that this difference could explain in part why men are more susceptible to developing AAA than women.

